# Expanding Access to Injectable Contraception: Results From Pilot Introduction of Subcutaneous Depot Medroxyprogesterone Acetate (DMPA-SC) in 4 African Countries

**DOI:** 10.9745/GHSP-D-17-00250

**Published:** 2018-03-21

**Authors:** Anna Stout, Siri Wood, George Barigye, Alain Kaboré, Daouda Siddo, Ida Ndione

**Affiliations:** a PATH, Seattle, WA, USA.; b PATH, Kampala, Uganda.; c UNFPA Burkina Faso, Ouagadougou, Burkina Faso. Currently with PATH, Dakar, Senegal.; d UNFPA Niger, Niamey, Niger.; e PATH, Dakar, Senegal.

## Abstract

PATH partnered with the United Nations Population Fund (UNFPA) and country ministries of health (MOHs) to coordinate pilot introductions of subcutaneous depot medroxyprogesterone acetate (subcutaneous DMPA or DMPA-SC, brand name Sayana Press) in Burkina Faso, Niger, Senegal, and Uganda from July 2014 through June 2016 in order to expand the range of methods available to women, particularly in remote locations. The pilot introductions aimed to answer key questions that would inform decisions about future investments in DMPA-SC and scaling up product availability and service-delivery innovations nationally. These questions included the extent to which DMPA-SC would appeal to first-time users of modern contraception, as well as adolescent girls and young women; whether DMPA-SC would add value to family planning programs or simply replace DMPA-IM or other modern methods; and the trends in injectables use when introducing DMPA-SC (or any injectable) at the community level for the first time. We implemented a multicountry monitoring system to track key indicators, including the number of doses administered by category of user (e.g., new users, by client age group) or delivery channel. Providers generally collected these data using their national programs' standard family planning registers. Data were analyzed for cumulative information and to examine trends over time using Microsoft Power Query for Excel and Tableau. Across the 4 countries, nearly half a million DMPA-SC doses were administered and approximately 135,000 first-time users of modern contraception were reached. Furthermore, 44% of the doses administered in 3 of the countries with data were to adolescent girls and young women under age 25. Switching from DMPA-IM to DMPA-SC was not widespread, ranging from 7% in Burkina Faso to 16% in Uganda. Results from these pilot introductions demonstrate that DMPA-SC has the potential to expand community-level access to injectables, maximize task-sharing strategies, and reach young women and new acceptors of family planning. Considered within the context of each country's setting, training approach, and introduction strategy, these results can help stakeholders in other countries make informed decisions about whether and how to include this contraceptive option in their family planning programs.

## Abstract (precis)

Nearly half a million doses of DMPA-SC were administered over 2 years in Burkina Faso, Niger, Senegal, and Uganda, with 29% of doses provided to first-time family planning users and 44% (in 3 countries) to adolescent girls and young women under age 25. Switching from intramuscular DMPA (DMPA-IM) was not widespread and generally decreased over time. Community health workers provided a higher proportion of DMPA-SC than DMPA-IM injections. Stock-outs in 2 countries hindered product uptake, highlighting the need to strengthen logistics systems when introducing a new method.

## INTRODUCTION

Worldwide, 214 million women would like to delay or stop childbearing but are not using any method of contraception.^1^ Sub-Saharan Africa has the lowest levels of contraceptive prevalence globally, with only 60% of demand for family planning satisfied.^2^ Evidence suggests that the addition of a new contraceptive method to the mix, or expanding geographic access to existing methods, attracts new contraceptive users and increases contraceptive prevalence.^3^^,^^4^

Injectable contraceptives are the most widely used modern method in sub-Saharan Africa, where their prevalence among married or in-union women is 10.7%—more than double that of oral contraceptive pills.^5^ In many African countries, however, injectables have not been made widely available outside of clinic settings due to restrictions on the cadres of providers that are authorized to administer injections. Community health workers (CHWs) often are not allowed to administer injections because of safety concerns. However, evidence from multiple countries shows that autonomous community-based distribution of injectable contraceptives by appropriately trained CHWs is safe, effective, and acceptable.^6^^,^^7^ The World Health Organization has called for such services to be part of comprehensive family planning programs,^7^ but despite this guidance, as of early 2017 only 11 countries in sub-Saharan Africa had enacted policies authorizing distribution of injectable contraceptives by CHWs.^8^ A new injectable contraceptive—subcutaneous depot medroxyprogesterone acetate (subcutaneous DMPA or DMPA-SC)—allows for easier administration than the traditional intramuscular DMPA (DMPA-IM) and offers the potential to overcome barriers to CHWs providing injectables.

The new subcutaneous DMPA injectable allows for easier administration than intramuscular DMPA and offers the potential to overcome barriers to CHWs providing injectables.

DMPA-SC is a 3-month, progestin-only injectable contraceptive administered into the fat below the skin. The most widely available DMPA-SC product, Sayana Press, is manufactured by Pfizer Inc. (Sayana Press is a registered trademark of Pfizer Inc.). As a lower-dose formulation and presentation of intramuscular DMPA, Sayana Press combines the drug and needle in the prefilled Uniject injection system. (Uniject is a trademark of Becton, Dickinson and Company.) Due to its simple presentation in a single device, DMPA-SC in Uniject requires less training for use than traditional intramuscular injections, making it especially suitable for task-sharing strategies—including administration by lay health workers in peripheral facilities and through community-based distribution.

**Figure:**
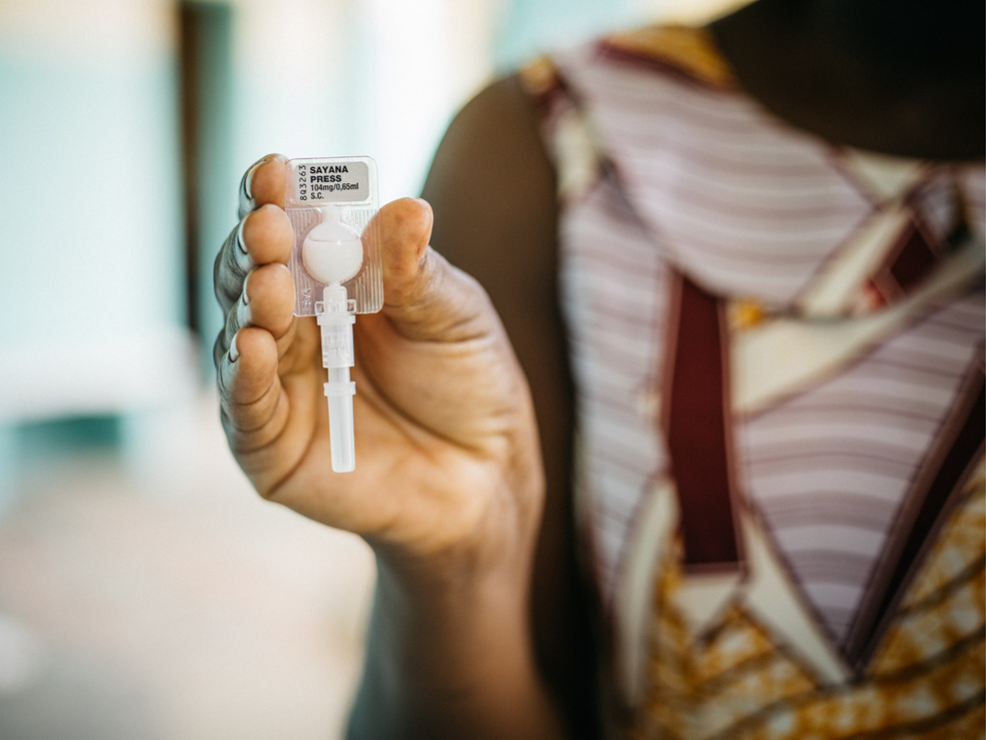
Sayana Press is a 3-month injectable contraceptive that is administered into the fat below the skin. © 2016 Gabe Bienczycki/PATH.

To date, all research studies of DMPA-SC in Africa have used the branded product, Sayana Press. Acceptability studies in Senegal and Uganda found that clients and providers alike preferred DMPA-SC to DMPA-IM due to quicker and easier administration, less pain with injection, and fewer side effects.^9^^,^^10^ Given the product's safety, acceptability, and ease of subcutaneous administration, ministries of health (MOHs) may be more likely to authorize community-based distribution of injectable contraception by CHWs and other community-level workers.^11^ DMPA-SC also offers the potential for self-administration.

At the 2012 London Summit on Family Planning, the governments of Burkina Faso, Niger, Senegal, and Uganda, among others, set ambitious objectives to increase contraceptive prevalence rates by 2020. Burkina Faso, Niger, and Senegal made commitments to support innovation in family planning service delivery by introducing DMPA-SC. Senegal and Uganda pledged to scale up community-based distribution, and Niger stated its intention to include injectables (DMPA-SC) in the range of methods offered by CHWs.^12^^–^^15^

In response to these commitments, PATH partnered with the United Nations Population Fund (UNFPA) and MOHs to coordinate pilot introduction of DMPA-SC in Burkina Faso, Niger, Senegal, and Uganda from July 2014 through June 2016. The MOHs leading these initiatives aimed to expand the range of methods available to women—particularly in remote locations—through informed choice counseling in order to reach additional users of modern contraception and to increase contraceptive prevalence rates. The pilot introductions offered injectable contraception to many communities for the first time, closer to where women live.

Global and national stakeholders had key questions about these pilot introductions to inform future investments in the product and decisions about scaling up product availability and service-delivery innovations nationally. These questions included the number of DMPA-SC doses that would be administered to clients during the pilot; the extent to which DMPA-SC would appeal to first-time users of modern contraception, as well as adolescent girls and young women; whether DMPA-SC would add value to family planning programs or simply replace DMPA-IM or other modern methods; and the trends in injectables use when introducing DMPA-SC (or any injectable) at the community level for the first time. To answer these questions, assess the reach of the pilot introductions, and inform mid-project course corrections, we developed a multicountry monitoring system across the 4 pilot countries. A set of global outcome indicators was established across country settings to allow collection and analysis of data in relation to different introduction strategies. A detailed report on design and implementation of this monitoring system, including lessons learned, is presented elsewhere.^16^ Results from the pilot introductions are described below and can help stakeholders in other countries make informed decisions on whether and how to include this contraceptive option in their family planning programs.

## PROJECT DESCRIPTION

### Planning

PATH engaged country governments early in the process of planning the pilot introductions in order to understand family planning goals and priorities and to assess interest in introducing the new contraceptive product, DMPA-SC. Key champions and supporters within the MOH were identified to provide leadership in designing the product introduction strategy and to provide technical and administrative oversight throughout the introduction process. Together, we drafted country-specific DMPA-SC introduction plans, validated the plans with a broader set of national family planning stakeholders, and established processes for MOH and NGO implementing partner engagement and coordination. Each country's introduction plan included an overview of the country's family planning landscape and goals and a description of the introduction strategy, including service-delivery channels (e.g., public or private sector, facility- and community-based delivery, and pharmacies or social marketing organizations); geographic areas for introduction; partners and their roles; product registration status, procurement, and distribution; provider training plans and communication and demand generation plans; and any monitoring, research, and evaluation activities.

Each country developed a country-specific subcutaneous DMPA introduction plan.

During the introduction planning process, MOH leadership in all 4 countries elected to introduce the product first at limited geographic scale. Burkina Faso and Senegal introduced DMPA-SC through delivery points at all levels of the health system, alongside DMPA-IM. They also implemented the pilots in 4 regions with the greatest population and highest rates of intention to use family planning, based on data from national Demographic and Health Surveys (DHS). In Senegal, a policy shift to allow community-based distribution of injectables at the outset of DMPA-SC introduction enabled CHWs to offer both DMPA-SC and DMPA-IM. Wanting to reach new users of family planning and expand geographic access for women living in remote areas, the Niger MOH elected to introduce DMPA-SC as the first offering of injectable contraception at the most peripheral facilities (health huts) in 2 districts, and through community-based distribution of the socially marketed product at the village level in 2 additional districts. In order to expand task sharing through community-based distribution, the Uganda MOH chose to introduce DMPA-SC alongside DMPA-IM in 28 districts where CHWs existed but were not consistently offering injectables. Table 1 presents a summary of the DMPA-SC pilot introductions in each of 4 African countries. The introduction period overall ran from July 2014 through June 2016, but each country had a different launch date. Burkina Faso launched in July 2014, Niger and Uganda in September 2014, and Senegal in January 2015; thus, some countries had shorter data collection periods.

**TABLE 1.** Overview of DMPA-SC Introduction and Provider Training Strategies by Country

	Burkina Faso	Niger	Senegal	Uganda
Product launch	July 2014	September 2014	January 2015	September 2014
Geographic scope	Over 680 public-sector facilities across the 4 most populous regions (23 rural, peri-urban, and urban districts)	211 public-sector community health huts in 2 rural districts; 50 CBD sites in 2 rural districts (4 districts total)	268 facilities and 637 health huts across the 4 most populous regions (31 rural, peri-urban, and urban districts)	CHWs linked to 336 public-sector health facilities across 28 rural and peri-urban districts
Service delivery channels	All levels of the health system, including public-sector mobile outreach from peripheral health and social promotion centers. Static NGO clinics and mobile outreach by NGO partners (MSI, ABBEF)	CHWs via public-sector health huts and private NGO CBD (ANIMAS-Sutura)	All levels of the health system, including by CHWs via health huts; NGO static clinics (MSI)	Public-sector CHWs; static NGO clinic and outreach in 1 site (Reproductive Health Uganda)
Community-based access to injectables	First offering of injectables through community outreach	First offering of injectables by CHWs at health huts and through CBD	Injectable provision previously authorized at health huts, though not widely available prior to DMPA-SC introduction	CBD of injectables previously authorized, though not widely available prior to DMPA-SC introduction
DMPA-SC and DMPA-IM offered side by side	Yes	No	Yes	Yes
Number of providers trained	∼1,900	∼300	∼2,000	∼2,100
Training approach	Rapid, cascade approach	Gradual, district-by-district approach	Rapid, cascade approach	Gradual, district-by-district approach

Abbreviations: ABBEF, Association Burkinabè pour le bien-être familiale; CBD, community-based distribution; CHW, community health worker; DMPA-IM, intramuscular depot medroxyprogesterone acetate; DMPA-SC, subcutaneous depot medroxyprogesterone acetate; MSI, Marie Stopes International.

### Coordination

PATH hired a national coordinator in each country to help guide country governments through essential steps in the project start-up phase, including tracking product registration, guiding the product introduction plans through the review and approval processes, and working with local experts to complete a quantification exercise, which informs the decision of how much product to order. The coordinators engaged in all aspects of project implementation including adapting training curricula, ensuring high-quality provider training, conducting field supervision, collecting and reporting monitoring data, and playing an active role in project steering committee meetings.

The MOH and all implementing partners in each country held routine coordination meetings to track DMPA-SC introduction progress, identify and respond to emerging challenges, and make decisions about program implementation. Some countries leveraged existing technical working groups for this purpose, while others convened a pilot project steering committee.

### Provider Training

Providers in each country were trained using DMPA-SC training materials developed by PATH.^17^ In a process led by the country coordinators, MOHs and implementing partners adapted this curriculum to fit their individual country context and providers' skill levels. Where relevant, countries also updated visual materials used by providers during client counseling sessions to include DMPA-SC.

An assessment of training needs was conducted to inform training plans in each country, including information on the number of providers to be trained, their cadres, and prior experience providing family planning counseling and injections or injectable contraception. The content and length of provider trainings varied by setting depending on the findings. For example, skilled providers needed training only in DMPA-SC and an injectable contraception refresher since they were already familiar with and offering other injectable contraceptives. Lower cadres of providers—such as CHWs—generally needed more complete training on administration of injectable contraception, and in some cases, training on the full range of available contraceptive methods.

In all countries, training content included theory and practice related to the provision of DMPA-SC. Providers were trained on the differences between DMPA-SC and DMPA-IM and how to counsel clients on both methods in the context of informed choice. Injection technique was first practiced on prosthetic models—the standard model being a condom filled with salt and tied off at the end—and was evaluated using an observational checklist included in the curriculum. Injection practice on the model was followed by a practicum where trainees administered injectable contraception—under the supervision of a qualified provider—to clients who had selected that method through informed choice counseling. Participants' injection technique was again evaluated using the same observational checklist with supervisors guiding them to master their technique.

Burkina Faso and Senegal implemented a rapid, cascade approach to training that worked well for introducing DMPA-SC at all levels of the health system in relatively large geographies. The MOHs and training partners in these countries organized centralized trainings for national master trainers, then regional trainings-of-trainers, followed by a cascade of trainings for district-level providers held simultaneously in each pilot introduction region. While implemented by partner NGOs, key staff from PATH or UNFPA and the MOH supervised all trainings. Unlike Burkina Faso and Senegal, introduction in Niger and Uganda was exclusively through CHWs and these countries followed a more gradual, district-by-district approach to training. In Niger, the MOH led the training, while in Uganda NGOs (PATH, Pathfinder International, FHI 360 and WellShare International) led the trainings, which were conducted in less centralized locations, closer to where CHWs live.

Burkina Faso and Senegal implemented a rapid, cascade training approach to introduce DMPA-SC, while Niger and Uganda followed a more gradual district-by-district training approach.

### Product Distribution

During the pilot introductions, DMPA-SC was distributed through the most appropriate mechanisms in each country, which involved one of 3 distinct approaches:

1. Integration of DMPA-SC into the existing public-sector national distribution systems from the outset (Burkina Faso and Niger)
2. Integration of DMPA-SC into a donor-funded initiative to improve distribution of contraceptive supplies and reduce stock-outs, called the Informed Push Model (Senegal)
3. Establishment of a parallel distribution system with donor funding, using a private distributor approved by the MOH as an alternative to the public-sector system (Uganda)

In the case of Uganda, the National Medical Stores (NMS) could not distribute the product only to the pilot districts before it was on the national Essential Medicines List, so PATH worked with subcontractor Uganda Health Marketing Group to establish a distribution approach for the pilot.

**Figure:**
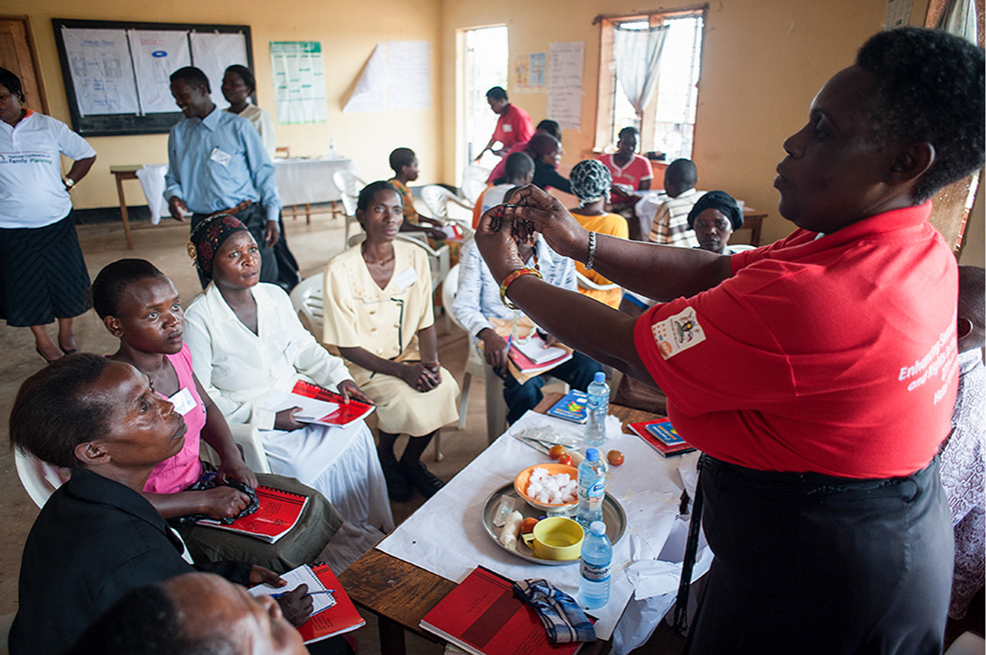
CHWs in Uganda received comprehensive training on all available family planning methods, including DMPA-SC. © 2014 Will Boase/PATH.

## METHODS

### Monitoring System Design

In collaboration with stakeholders from each country, we employed a 4-phase approach to monitoring DMPA-SC pilot introduction^16^:

1. Develop and define global indicators
2. Integrate global indicators into country data collection tools
3. Facilitate consistent reporting and data management
4. Analyze and interpret data and share results

We selected global outcome indicators based on key areas of interest to country stakeholders and donors—for example, the potential for DMPA-SC to expand family planning access for women who had never used family planning, the potential to reach young women, and the extent to which current users of other modern contraceptive methods switched to DMPA-SC. Monitoring indicators represent high-level data on family planning service delivery, counting the number of doses administered by category of user (e.g., new users, client age group) or delivery channel (e.g., community-based distribution). Global indicators were vetted with MOHs and local implementing partner organizations and refined accordingly. Table 2 lists the global pilot project indicators and the purpose of each indicator. Other indicators were also monitored but are not reported here, as they do not bear as directly on our objectives—for example, the types of providers trained and the number of DMPA-SC doses distributed to health facilities. More detailed information about the global indicator definitions, data requirements, and measurement levels is available elsewhere.^18^

**TABLE 2.** Global Monitoring Indicators for Pilot Introduction of DMPA-SC

Global Monitoring Indicator	Purpose
Number of DMPA-SC doses administered to clients	Documents the number of DMPA-SC doses administered to clients, independent from other injectable products Provides the denominator for indicators on new users, switching from DMPA-IM, and switching from other modern methods
Number and percentage of DMPA-SC doses administered to first-time users of modern contraception (“new users”)^a^	Indicates the total number of new users of modern contraception reached with DMPA-SC and the share of total DMPA-SC doses administered to first-time users, by health system level where relevant Helps determine the extent to which the product is reaching new users, as opposed to users who had previously used another modern method The denominator for the percentage indicator is the number of DMPA-SC doses administered to clients
Number and percentage of DMPA-SC doses administered to clients under age 20, to those ages 20 to 24, and those ages 25 and older (Niger, Senegal, and Uganda only)	Documents the extent to which providers administer DMPA-SC doses to adolescent girls and young women May indicate whether DMPA-SC is an attractive method choice for adolescents and younger women May highlight areas where additional training on provision of family planning methods (and/or injectables) to youth could be needed The denominator for the percentage indicator is the sum of doses administered to clients in each age category
Number and percentage of DMPA-SC doses administered to clients who switched from DMPA-IM (Burkina Faso, Senegal, and Uganda only)	Documents the number and proportion of DMPA-SC doses administered to clients switching from DMPA-IM, in order to track an early concern of stakeholders that DMPA-SC—a more expensive product at the time—would potentially replace DMPA-IM May indicate whether women and/or providers prefer DMPA-SC to DMPA-IM May indicate the need to follow up with providers during supervision to ensure DMPA-SC is not promoted as a replacement for DMPA-IM The denominator for the percentage indicator is the number of DMPA-SC doses administered to clients
Number and percentage of DMPA-SC doses administered to clients who switched from modern methods other than DMPA-IM (Burkina Faso and Senegal only)	Documents the number of DMPA-SC doses administered to clients switching from modern methods other than DMPA-IM The denominator for the percentage indicator is the number of DMPA-SC doses administered to clients
Number of DMPA-IM doses administered to clients	Documents the volume of DMPA-IM doses administered to clients, independent from other injectable products Provides input for the numerator and denominator for the indicator on relative proportions of DMPA-SC and DMPA-IM administered, by level
Relative proportions of DMPA-SC and DMPA-IM administered, by level (where both methods are available)	Documents the relative share of the market comprised of DMPA-SC and of DMPA-IM, by level, where providers offer both methods; may indicate the preference of women and/or providers for each method, though factors such as provider skill level and potential bias should also be considered Numerators include the number of doses of DMPA-SC and DMPA-IM administered to clients; the denominator is the sum of the number of doses of DMPA-SC and DMPA-IM administered to clients
Number and percentage of facilities with a stock-out of DMPA-SC	Documents the extent of DMPA-SC stock-outs and contextualizes trends in DMPA-SC consumption and in the overall method mix Helps identify locations where the distribution system and/orfacility stock management practices may require reinforcement The denominator for the percentage indicator is the number of facilities active in the provision of DMPA-SC that reported during the same period
Number of facilities active in the provision of DMPA-SC that reported this period	Documents the number of facilities that reported on DMPA-SC in a given period Provides input on data completeness Provides the denominator for the percentage of facilities with a stock-out of DMPA-SC

Abbreviation: DMPA-SC, subcutaneous depot medroxyprogesterone acetate.

a A first-time user of modern contraception—also referred to as “new user”—is defined as a client who has elected to use a modern method of contraception for the first time in her life.

### Data Collection and Analysis

Providers generally collected service delivery data using their national programs' standard family planning registers. New registers were developed where injectables were previously not available or where service delivery data were not tracked, such as community-based distribution by CHWs in Uganda. To ensure that data were comparable within and across country settings, providers were trained on the correct and consistent application of indicator definitions. This approach ensured existing country data collection systems were leveraged for pilot project data collection, with one exception. In Senegal, it was not possible to track all of the global indicators through routine data collection and reporting practices, so we established a “sentinel site” system. Under this system, data for new users, age, and method switching were collected from anon-representative sample of 35 health facilities selected from the country's pilot regions.

National health information systems (HIS) data generally reach the central level every 6 months or less often and do not disaggregate injectables by type (e.g., DMPA-IM vs. DMPA-SC). Because stakeholders desired quarterly disaggregated data to track progress and inform programmatic course corrections, relying on HIS data was not feasible for this project. Instead, monitoring focal persons for the pilot project collected data each quarter directly from the district or facility level—including sentinel sites—using tools specifically developed for the project. Some NGO implementing partners were already reporting their service delivery data to the MOH at the district level, which supported a smooth flow of data under the pilot introduction. Others reported data directly to designated monitoring focal points. Service delivery data related to this project were also reported to each country's existing HIS under national standard operating procedures.

**Figure:**
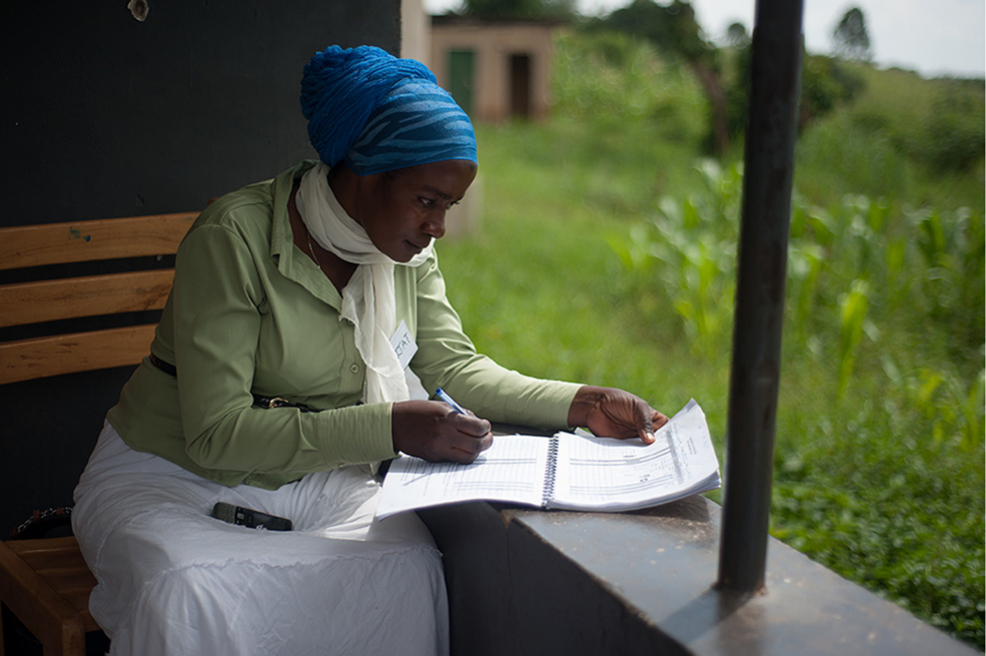
A CHW in Uganda enters service delivery data into a family planning register. © 2014 Will Boase/PATH.

We cleaned and analyzed the service delivery data from all 4 countries using Microsoft Power Query for Excel and Tableau. Indicator data were analyzed for cumulative information and to examine trends over time. The impact of some indicators on others was assessed, such as the impact of stock-outs on product consumption and doses administered to first-time users of modern contraception. As a secondary analysis, monitoring data on DMPA-SC use by client age group were compared with data on injectable use by age group from the DHS in selected countries in order to evaluate adolescent access to DMPA-SC via pilot delivery channels. Monitoring data do not include personal identifiers and thus cannot track individual users over time. Indicators on age, new users, and method switching refer to a proportion of doses administered to users in each category. “Total DMPA-SC doses administered” is the denominator for several calculated monitoring indicators (e.g., new users, age, method switching). Data visualizations were validated by project country teams, who provided further contextual information to help interpret the data prior to disseminating results to donors and more broadly to global and national stakeholders.

## RESULTS

### Product Consumption and Stock-Outs

**FIGURE 1.**
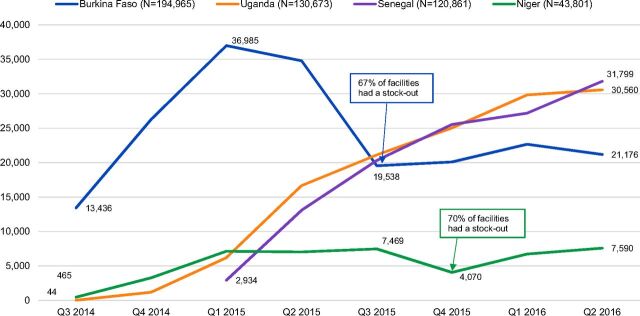
Number of DMPA-SC Doses Administered by Quarter and Country, 2014–2016 Abbreviations: DMPA-SC, subcutaneous depot medroxyprogesterone acetate; Q, quarter.

From July 2014 through June 2016, nearly half a million doses of DMPA-SC were administered across the 4 pilot introduction countries, with 2 countries experiencing periods of heavy DMPA-SC stock-outs. Overall, providers in Burkina Faso administered the highest number of DMPA-SC doses—194,965—during the introduction period, from July 2014 through June 2016 (Table 3). In Burkina Faso, consumption increased rapidly during the first 2 quarters of introduction but was substantially hampered by stock-outs from the second quarter through the fourth quarter of 2015. Stock-outs peaked in September 2015, when 67% of facilities reported a stock-out of DMPA-SC. Two primary factors—large quantities of product expiring and poor weekly stock surveillance—contributed to stock-outs. Consumption increased only modestly in 2016 as stock-outs slowly began to resolve (Figure 1). Family planning clients procured 94% of DMPA-SC doses from public-sector facilities and 6% through private NGO delivery.

**TABLE 3.** Cumulative Results From Pilot Introduction of DMPA-SC Across 4 Countries, 2014–2016

	Burkina Faso	Niger	Senegal	Uganda
No. of DMPA-SC doses administered	194,965	43,801	120,861	130,673
% of DMPA-SC doses administered to new users	25	42	24^a^	29
% of DMPA-SC doses administered to adolescent girls and young women				
<20 years old	-	16	8^a^	12
20–24 years old	-	34	26^a^	32
<25 years old	-	50	34^a^	44
% of DMPA-SC doses administered to clients switching from:				
DMPA-IM	7 (8 public; 2 NGO)	-	13^a^	16
Other modern methods besides DMPA-IM	17 (18 public; 9 NGO)	-	12^a^	-
Any modern method	24 (26 public; 11 NGO)	-	25^a^	-
Proportion of DMPA-SC relative to DMPA-IM where offered in parallel				
At the community level^b^	-	-	72	75
At all levels	16	-	30	-
% of health facilities reporting a stock-out of DMPA-SC (highest reported)	67	70	<2	9

Abbreviations: DMPA-IM, intramuscular depot medroxyprogesterone acetate; DMPA-SC, subcutaneous depot medroxyprogesterone acetate.

- Data not available.

a Senegal data derived from the sentinel sites.

b DMPA-SC available only at the community level in Niger and Uganda.

Over 2 years, nearly half a million doses of subcutaneous DMPA were administered across the 4 countries.

In Senegal, providers administered 120,861 doses of DMPA-SC from January 2015 through June 2016. As in Burkina Faso, consumption increased rapidly during the first 2 quarters of introduction. Providers in Senegal administered the highest number of doses (31,799) of all countries during the final reporting period (i.e., second quarter of 2016) (Figure 1). Stock-outs in Senegal were insignificant, with less than 2% of facilities reporting a stock-out at any point under the privately funded distribution system—the Informed Push Model. The public sector was the primary source of DMPA-SC, where 97% of all reported administered doses were procured.

In Uganda, where DMPA-SC was available only through community-based distribution, CHWs administered 130,673 doses of DMPA-SC from September 2014 through June 2016, with steady growth in consumption over the duration of pilot introduction. Stock-outs in Uganda were negligible, with 3% or less of facilities reporting a stock-out under the privately funded distribution system managed by Uganda Health Marketing Group (UHMG)—except in the final month of pilot introduction, when 9% of facilities experienced a stock-out due to delays in the arrival of a new product order.

CHWs in Niger administered 43,801 total doses—39,957 at public-sector health huts in 2 districts and 3,844 through NGO-led community-based distribution in 2 additional districts—from September 2014 through June 2016. The public sector was the primary driver of consumption, with 91% of doses administered via health huts. Consumption increased steadily during the first 2 quarters of introduction before plateauing and ultimately declining due to stock-outs, which peaked in November 2015 when 70% of facilities reported a stock-out of DMPA-SC (Figure 1). Modest gains in consumption were experienced during the first and second quarters of 2016 as stocks in health hut were replenished.

### Reaching New Users of Modern Contraception

#### Overall

Across the 4 countries, DMPA-SC was administered to an estimated 135,000 women using modern contraception for the very first time (29% of all doses administered were to new users). The share of DMPA-SC administered to new users was greatest in Niger, where it constituted 42% of all doses administered. In Uganda, where DMPA-SC was available only through community-based distribution, 29% of all doses administered were to new users. Approximately one-quarter of doses administered in both Burkina Faso and Senegal went to new users (Table 3). DMPA-SC was available at all levels of the health system in these 2 countries compared with Niger and Uganda, where it was available exclusively from CHWs at remote health huts and through community-based distribution. Data on new users in Senegal were derived from the sentinel sites, which represented 7.5% of overall product consumption in Senegal. While the sentinel site data cannot be generalized to the entire introduction initiative, they provide valuable insights into pilot introduction in Senegal.

29% of all subcutaneous DMPA doses administered across the 4 countries were to new contraceptive users.

#### Trends

**FIGURE 2.**
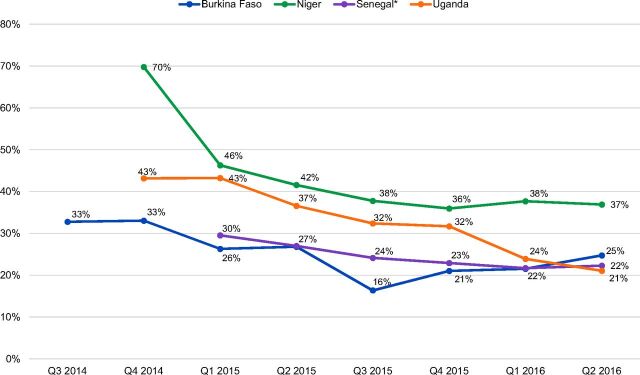
Proportion of DMPA-SC Doses Administered to New Users by Quarter and Country, 2014–2016 Abbreviations: DMPA-SC, subcutaneous depot medroxyprogesterone acetate; Q, quarter. * Senegal data derived from the sentinel sites.

The proportion of doses administered to new users ranged between 30% (Senegal, sentinel sites) and 70% (Niger) across the 4 countries during the first full quarter of pilot introduction and declined gradually over time as women returned for reinjections (and the denominator—total doses administered—increased) (Figure 2). The main exception to this trend was Burkina Faso, where stock-outs resulted in a smaller proportion of doses administered to new users during the third quarter of 2015. During times of low or no stock, providers in Burkina Faso reserved DMPA-SC units for continuing injectable users, as they were reluctant to start new users on a method that might not be available at the client's next appointment. The proportion of new users began to return toward previous levels beginning in the fourth quarter of 2015 as stock-outs began to resolve.

As noted, doses administered to women returning for reinjections of DMPA-SC contributed to the “total doses administered” denominator, and thus to the declining proportions of doses administered to new users (and those switching from other methods) over time. Reinjections of DMPA-SC were not selected as a global indicator as this data is generally challenging to collect; however, data on doses administered to repeat users of DMPA-SC were collected at sentinel sites in Senegal and were calculated from Burkina Faso data. In these 2 countries, we observed similar trends of doses administered to returning clients. By the third quarter of pilot introduction, half of all DMPA-SC doses administered were to clients receiving repeat injections, and this proportion increased to two-thirds in the final 2 quarters of the pilot. Overall, in both Burkina Faso and Senegal, half of all doses administered were to clients receiving repeat injections of DMPA-SC.

### Access to Injectable Contraception forAdolescent Girls and Young Women

In aggregate across Niger, Senegal (sentinel sites), and Uganda, 44% of DMPA-SC doses administered were to adolescent girls and young women under age 25. Approximately 12% of doses administered were to adolescent girls and young women under age 20, and 32% were to those ages 20 to 24. Comparing across countries, the proportion of doses administered to women under age 25 was higher in Niger (50%) and Uganda (44%) compared with Senegal (34%) (Table 3). The distribution of doses administered by client age group remained relatively constant in each country over the course of introduction. Data on doses administered by client age group were not collected in Burkina Faso.

44% of DMPA-SC doses administered across Niger, Senegal, and Uganda were to adolescent girls and young women under age 25.

**FIGURE 3.**
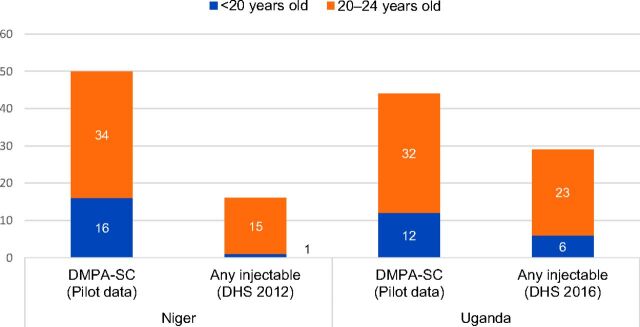
Percentage of Clients in Niger and Uganda Accessing DMPA-SC During the Pilot Compared With Women Reporting Use of Any Injectable in the DHS, by Age Group Abbreviations: DHS, Demographic and Health Survey; DMPA-SC, subcutaneous depot medroxyprogesterone acetate.

We also compared pilot data on DMPA-SC use by client age group with data on any injectable use by age group from the DHS in Niger and Uganda. We observed that a higher proportion of women under age 25 accessed DMPA-SC in the pilot compared with any injectable in the DHS. In Uganda, of the women who reported using any injectable in the DHS (primarily DMPA-IM), 29% were under age 25 (6% were under age 20 while 23% were ages 20 to 24).^19^ By comparison, of the doses administered to women using DMPA-SC in the pilot, 44% were to women under age 25 (12% were under age 20 and 32% were ages 20 to 24). A similar trend was observed in Niger, where of the women who reported using any injectable in the 2012 DHS, 16% were under age 25 (1% were under 20 while 15% were ages 20 to 24).^20^ By comparison, of the DMPA-SC doses administered to women in the pilot, 50% were to women under age 25 (16% were under 20 and 34% were ages 20 to 24) (Figure 3). DHS data are from a representative, population-based sample whereas pilot data are solely from the regions and delivery channels relevant to each country's introduction strategy—in this case, community-based distribution of injectables by CHWs in Uganda and CHW delivery of DMPA-SC through rural health huts in Niger.

### Method Switching

#### Cumulative

Over the course of pilot introduction in the 4 countries, the number of DMPA-SC doses administered to clients switching from DMPA-IM and from other modern methods was tracked. Cumulatively, switching from DMPA-IM to DMPA-SC was highest in Uganda, where 16% of all DMPA-SC doses administered were to women switching from DMPA-IM (Table 3). Switching from DMPA-IM made up 13% of doses administered in Senegal (sentinel sites) and 7% in Burkina Faso, where switching was higher in the public sector (8%) compared with the NGO sector (2%). Data on method switching were not collected in Niger, where switching from DMPA-IM would theoretically be low, as DMPA-IM was not offered in parallel with DMPA-SC during pilot introduction.

Of the countries with relevant data, the proportion of DMPA-SC administered to women switching from modern methods other than DMPA-IM was 12% in Senegal (all levels, sentinel sites) and 17% in Burkina Faso, where switching was again higher in the public sector (18%) than the NGO sector (9%).

#### Trends

**FIGURE 4.**
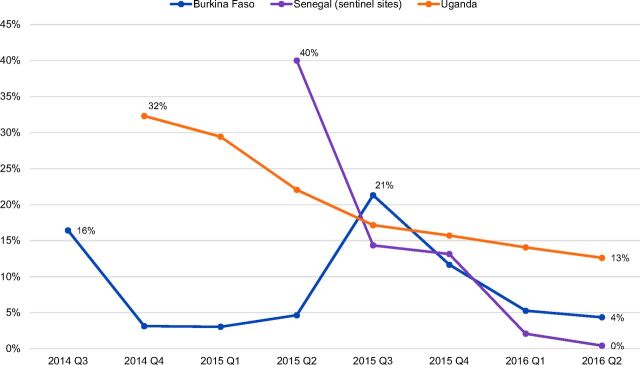
Proportion of DMPA-SC Doses Administered to Clients Switching From DMPA-IM, by Quarter and Country, 2014–2016 Abbreviations: DMPA-IM, intramuscular depot medroxyprogesterone acetate; DMPA-SC, subcutaneous depot medroxyprogesterone acetate; Q, quarter.

In general, the proportion of doses administered to women switching to DMPA-SC from DMPA-IM and other modern methods declined over time across country settings. In Burkina Faso, the proportion of DMPA-SC administered to women switching from DMPA-IM fell from 16% during the third quarter of 2014 to 4% during the second quarter of 2016, but rose briefly during the third quarter of 2015 due to switching related to stock-outs (Figure 4). When DMPA-SC was unavailable, DMPA-SC clients were switched temporarily to DMPA-IM and then switched back to DMPA-SC as stock-outs resolved, resulting in a spike in switching. In Senegal, switching to DMPA-SC from DMPA-IM fell from 40% during the second quarter of 2015 to less than 2% during the second quarter of 2016 (sentinel sites). In Uganda, switching to DMPA-SC from DMPA-IM declined gradually over time, from 32% during the fourth quarter of 2014 to 13% during the second quarter of 2016. In certain regions of Burkina Faso and Senegal, switching was higher early on in introduction due to a misconception among providers that DMPA-SC was intended to replace DMPA-IM.

In Burkina Faso, switching from modern methods other than DMPA-IM fell from 44% during the third quarter in 2014 to 5% during the second quarter of 2016. In Senegal, switching from other modern methods decreased from 19% during the second quarter in 2015 to 7% during the second quarter of 2016 (sentinel sites).

### DMPA-SC's Share of the DMPA Market

#### Cumulative

**FIGURE 5.**
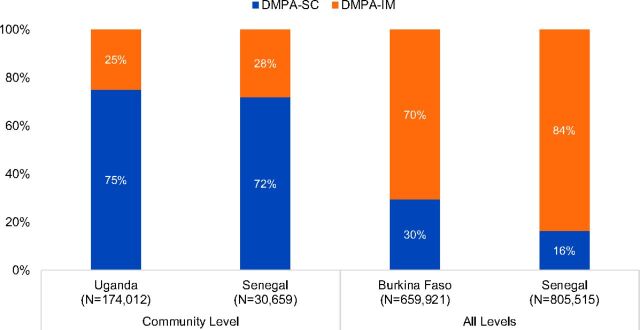
Relative Proportions of DMPA-SC and DMPA-IM Administered by Level of the Health System and Country,^a^ 2014–2016 Abbreviations: DMPA-IM, intramuscular depot medroxyprogesterone acetate; DMPA-SC, subcutaneous depot medroxyprogesterone acetate. ^a^No data available from the community level in Burkina Faso. No data available for Niger.

Data on the relative proportions of DMPA-SC and DMPA-IM are not available from Niger or from the community level in Burkina Faso, where DMPA-IM was not offered alongside DMPA-SC. Where the 2 methods were offered in parallel, DMPA-SC comprised approximately three-quarters of all injectables administered at the community level (i.e., in Senegal and Uganda). By comparison, DMPA-SC made up only 16% and 30% of injectables when administered across all levels of the health systems in Senegal and Burkina Faso, respectively (Figure 5, Table 3). In Burkina Faso, the relative share of DMPA-SC was higher in the NGO sector—which had a greater focus on outreach—at 46%, compared with the public sector, at 29% (data not pictured).

#### Trends

The proportion of DMPA-SC relative to DMPA-IM typically increased gradually over time as introduction advanced in each country. In Senegal, the share of DMPA-SC increased from 2% during the first quarter of 2015 to 21% during the second quarter of 2016 at the facility level, and from 21% during the first quarter of 2015 to 74% during the second quarter of 2016 at community-level health huts. In Uganda, the share of DMPA-SC increased steadily from 53% during the fourth quarter of 2014 to 79% during the fourth quarter of 2015, where it held constant through the remainder of the pilot (through the second quarter of 2016).

In Burkina Faso, the share of DMPA-SC relative to DMPA-IM fluctuated considerably across all levels of the health system over the course of the 2-year pilot due to intermittent stock-outs of DMPA-SC. The share of DMPA-SC increased from 29% of injectables during the third quarter of 2014 to 38% during the first quarter of 2015, yet decreased to only 20% during the fourth quarter of 2015 when stock-outs neared their peak. In Burkina Faso's NGO sector, the relative share of DMPA-SC increased steadily from 34% during the third quarter of 2014 to 60% during the second quarter of 2015, before decreasing due to stock-outs.

## DISCUSSION

Data from 2 years of pilot introduction indicate that DMPA-SC has the potential to add value to national family planning programs by expanding the range of methods available through community-based distribution in order to reach new acceptors of family planning, as well as to reach adolescent girls and young women. Switching to DMPA-SC from DMPA-IM and other modern methods was not widespread, further underscoring the potential appeal of DMPA-SC to new users. The negative impact of stock-outs on product consumption and addition of new users highlights the need to strengthen existing commodity distribution systems when introducing a new method. Considered within the context of each country's setting, training approach, and pilot introduction strategy, these monitoring results can offer guidance for other settings on whether and how to introduce DMPA-SC or other new contraceptive methods into national family planning programs.

Subcutaneous DMPA has the potential to add value to national family planning programs by expanding the range of methods available through community-based distribution in order to reach new family planning acceptors, adolescents, and young women.

Results from the 4 pilot introductions are best understood in the context of each country's unique introduction strategy, training approach, and product launch timing. Monitoring data on total DMPA-SC doses administered demonstrate that high consumption volumes can be achieved through public- or multisector product introduction at all levels of the health system, as done in Burkina Faso and Senegal. The MOHs of these countries chose to introduce DMPA-SC at all levels of the health system alongside DMPA-IM to expand the range of available contraceptive methods and maximize client choice. Using a rapid, cascade approach to provider training ensured that the maximum number of providers were trained in the shortest time possible, contributing to swift product uptake. The high consumption volume in Burkina Faso was also a result of their launching the product before other countries (thus having a longer time for administering doses) and introducing the product in the country's 4 most populous regions.

Data from Niger—where the pilot represented the first offering of injectables at the community level and where nearly half of doses administered were to new users—make a particularly compelling case for extending access to injectable contraceptives to areas where they were not previously available. Relatively high consumption volumes can be achieved through community-level delivery, especially when making injectables available at this level for the first time or addressing high unmet need. The consumption volume achieved in Niger's public sector is significant considering the primary introduction channel consisted of low-volume health huts in only 2 rural districts.

Community-based distribution programs are a potential strategy to reduce unmet need in countries with large rural populations.^4^ Beyond the methods they provide directly, CHWs can also help increase use of clinic-administered contraceptive methods through counseling and referrals.^4^ Task sharing can increase contraceptive access even further by expanding the range of methods CHWs can offer. When CHWs provide contraceptives directly, uptake is significantly greater than when they offer referrals alone.^21^^,^^22^ Monitoring data on the relative proportions of DMPA-SC and DMPA-IM administered at the community level versus at all levels of the health system reveal that DMPA-SC is well positioned for task-sharing strategies. In Senegal and Uganda, a higher relative proportion of DMPA-SC doses were administered at the community level (75%) compared with all levels of the health system in Senegal and Burkina Faso (16% and 30%, respectively). Two factors may account for the greater use of DMPA-SC at the community level. First, CHWs are often more comfortable administering DMPA-SC than DMPA-IM. They find it easier to prepare and inject due to its all-in-one presentation in Uniject, compared with the separate vial and syringe used for DMPA-IM.^7^ In addition, previous studies on the acceptability of DMPA-SC among DMPA-IM users and providers revealed that the majority of clients preferred DMPA-SC due to the shorter needle, less pain during injection, and fewer reported side effects,^9^^,^^10^ perceptions that spread by word of mouth during pilot introduction, further adding to the method's appeal.

### Stock-Outs Negatively Affect Product Consumption

Stock-outs had a negative effect on both consumption volumes and new users in Burkina Faso and Niger—underscoring the importance of commodity security and strengthening existing distribution systems when introducing a new contraceptive product. Large quantities of product expiring, in addition to poor weekly stock surveillance, contributed to stock-outs in these 2 settings. Stock-outs were insignificant in Senegal and Uganda, where distribution was managed by private distribution partners. The impact of stock-outs was far-reaching and reverberated beyond periods of inadequate supply. Consumption trends in Burkina Faso and Niger revealed that product use was slow to return to previous levels following a period of heavy stock-outs, perhaps due to lack of provider and/or client confidence in continued product availability. Research on malaria treatment has demonstrated that stock-outs have the potential to alter prescribing behavior of providers.^23^ Inadequate supply of drugs and the fear of stock-outs have led providers to avoid prescribing certain drugs or to ration drugs for patients they perceive as most in need or most deserving.^24^ To design effective supply chains for community-based distribution programs, it is important to involve CHWs in the process,^25^ consider CHW literacy levels, determine ways to track logistics management information systems, and track and aggregate data.^26^

### DMPA-SC Use by Adolescents and Young Women

Pilot introduction data on the proportion of DMPA-SC doses administered by client age group indicate that DMPA-SC may be an attractive option for adolescent girls and young women, particularly where available at the community level. Of the women using DMPA-SC in the pilot, a higher proportion were under age 25 compared with women using any injectables in the Niger and Uganda DHS. DHS data are from a population-based sample, whereas pilot data are solely from community-based distribution of injectables in 28 districts in Uganda, and health hut delivery of DMPA-SC by CHWs in 2 districts in Niger. However, the comparison indicates that youth may feel more comfortable or be better able to access contraceptives privately from a CHW in their own village. Since injectables were not previously available at health huts in Niger, young rural women interested in injectable contraception would have been required to travel some distance to a referral facility. These findings support existing evidence that community-based outreach is an effective intervention for increasing contraceptive use among youth.^27^ The new option offers greater convenience and does not require travel or time away from home— an added benefit for women whose mobility is restricted by sociocultural norms.

### Switching From DMPA-IM to DMPA-SC Not Widespread

Offering multiple methods provides individuals who find their initial choice unacceptable with the opportunity to switch methods—which may reduce method-specific continuation but improves client satisfaction and overall contraceptive continuation.^28^ Positioning DMPA-SC and DMPA-IM side by side may improve injectables continuation by making another injectable option available to clients, particularly when one method is stocked out. Although introduction of DMPA-SC prompted some existing contraceptive users to discontinue their current method in favor of DMPA-SC, switching to DMPA-SC from other modern methods was not widespread and generally decreased gradually over time. In certain settings, switching was higher initially due to a misconception among providers that DMPA-SC was intended to replace DMPA-IM. This assumption was corrected through ongoing field supervision. Monitoring data from Uganda show that no more than 16% of total DMPA-SC doses administered went to women switching from DMPA-IM, and no more than 17% in Burkina Faso—indicating that DMPA-SC adds value to family planning programs rather than simply replacing existing methods. As communities become fully aware of a new method over time, the downward trend in switching is likely to continue. Switching to DMPA-SC from DMPA-IM and other modern methods was higher in Burkina Faso's public sector compared with the NGO sector, likely due to NGOs' focus on long-acting methods such as the implant and the intrauterine device (IUD), as women already using long-acting methods may be less likely to switch to an injectable.

Switching from intramuscular DMPA or other modern methods to subcutaneous DMPA was not widespread.

In Uganda, where the relative proportion of DMPA-SC through community-based distribution was high (75%) compared with DMPA-IM (25%), the number of DMPA-IM doses administered stayed relatively constant over time—while DMPA-SC consumption increased steadily—indicating the presence of a consistent DMPA-IM client base that did not switch methods. In Senegal and Burkina Faso, where DMPA-SC was made available at all levels of the health system, the relative proportion of DMPA-IM was much higher (70% and 84%, respectively) than DMPA-SC (16% and 30%, respectively). DMPA-IM had been widely available and widely used among family planning clients in both of these settings, and those clients generally stayed with DMPA-IM. Nevertheless, the proportion of DMPA-SC relative to DMPA-IM increased gradually across the 3 countries where consumption was not affected by stock-outs. Based on global agreements and MOH guidance at the outset of these pilot introductions, project coordinators conveyed the message to providers (throughout training and follow-up supervision in all 4 countries) that DMPA-SC was not intended to replace DMPA-IM. If replacement becomes a clear priority of global and national family planning stakeholders, the proportion of DMPA-SC would likely increase.

### Limitations

Due to the absence of client identifiers in monitoring data, individual clients were not tracked over time, resulting in certain limitations on analysis; for example, we were not able to distinguish how many doses were given to the same woman, since reinjection every 3 months is necessary for anyone continuing to use this method. Indicators on doses administered by client age group and method switching refer only to a proportion of doses administered to users in each category from quarter to quarter and do not reveal the true proportion of *individual clients* in each of those categories over time. For example, a client who is 20 years of age and receives 4 doses of Sayana Press over the course of a year has her age counted at each visit. “Total DMPA-SC doses administered” is the denominator for several calculated monitoring indicators (e.g., new users, age, method switching), which contributes to these indicators declining over time as clients return for repeat injections.

Reported figures underestimate actual product consumption because no country reporting was 100% complete. For example, overall, an average of 80% of health structures reported in Burkina Faso; 93% in Niger; 90% in Uganda (where health facilities reported aggregated CHW data); and 98% and 97% for routine reporting and sentinel sites, respectively, in Senegal. Due to the absence of formalized, contractual relationships, limited data were shared from NGO partners in Niger, Senegal, and Uganda—making it difficult to calculate the impact of introduction in the private NGO sector in these settings. In Burkina Faso, NGO-sector reporting was integrated with the public-sector HIS prior to the start of pilot introduction, which aided in accounting for NGO-sector distribution and allowed disaggregation by sector for certain indicators. For other indicators, however, the aggregation of public and private NGO-sector data at the district level, as well as inaccurate application of indicator definitions, prevented disaggregation by sector or delivery channel (e.g., community-based distribution).

## SCALING UP INTRODUCTION OF DMPA-SC

Using monitoring data to guide decision making, all 4 pilot countries decided to scale up provision of DMPA-SC nationwide, with expansion underway. In most cases, 1 year of monitoring data provided an adequate evidence base to inform the country MOH's decisions about scale. For example, the high proportion of doses administered to first-time users of modern contraception through rural health huts in Niger helped the MOH decide to scale up availability of injectables at health huts nationwide in order to expand the range of methods available to women through informed choice counseling—particularly in remote locations—in order to reach additional users of modern contraception and increase modern contraceptive prevalence rates. The Pfizer price agreement to offer DMPA-SC at US$1 per dose to qualified purchasers was another significant factor in these decisions.^29^ As of May 2017, the price dropped even further to $0.85,^30^ nearly equivalent to the cost of DMPA-IM at $0.83 ($0.74 for the vial^31^ and $0.09 for the syringe^32^). Monitoring results from pilot introduction of DMPA-SC may prove particularly beneficial for countries planning to introduce DMPA-SC or other new contraceptive methods and can inform country decision making about contraceptive introduction strategies.

All 4 countries decided to scale up provision of subcutaneous DMPA nationwide based on results of the pilot introductions.

## Notes

**Peer Reviewed**

**Funding:** This work was supported by the Bill & Melinda Gates Foundation, Seattle, WA [award number OPP1060986]; and the Children's Investment Fund Foundation, London, United Kingdom [Request #333]. The funding sources did not play a role in study design; the collection, analysis, and interpretation of data; the writing of the report; or the decision to submit the article for publication. All data created during this research are openly available from Harvard Dataverse at https://doi.org/10.7910/DVN/PQ7IUE.
